# Cardiovascular safety of febuxostat and allopurinol in patients with gout: A meta-analysis

**DOI:** 10.3389/fphar.2022.998441

**Published:** 2022-09-30

**Authors:** Xudong Guan, Shengzhao Zhang, Jiayan Liu, Fengbo Wu, Lingyan Zhou, Ying Liu, Na Su

**Affiliations:** ^1^ Department of Pharmacy, West China Hospital, Sichuan University, Chengdu, China; ^2^ West China School of Pharmacy, Sichuan University, Chengdu, China; ^3^ Department of Pharmacy, Karamay Central Hospital, Xinjiang, China; ^4^ Department of Dermatology and Venereal Disease, West China Hospital, Sichuan University, Chengdu, China

**Keywords:** gout, allopurinol, febuxostat, cardiovascular safety, an individual-patient data level META analysis

## Abstract

**Background:** Gout is a common disease and is usually treated with uric acid-lowering drugs (the most commonly used of which are febuxostat and allopurinol). However, the cardiovascular safety of febuxostat and allopurinol is still controversial. The purpose of our study is to evaluate the cardiovascular safety of the two drugs in patients with gout using one-stage and two-stage meta-analysis.

**Methods:** PubMed, Embase, CBM, CNKI, WanFang, Central, and VIP were searched from inception to 30 January 2022. Randomized controlled trials which evaluated the cardiovascular safety of febuxostat or allopurinol for treating patients with gout were included. Based on the Kaplan–Meier curves of the two studies, individual patient data (IPD) were extracted and reconstructed. We used time-varying risk ratios (RRs) to summarize time-to-event outcomes, and the RRs of MACE incidence, cardiovascular mortality, and all-cause mortality were calculated by a multi-level flexible hazard regression model in 1-stage meta-analyses. *p* values were calculated using a log-rank test. At the same time, using the reconstructed IPD, we performed 2-stage meta-analyses to inform the quantitative estimates of time-specific relative risks at the six time points (1 , 2, 3, 4, 5, and 6 years) based on a random-effects model.

**Results:** Two RCTs with 12,318 participants were included. In the incidence of major adverse cardiovascular events between the two regimens, there was no significant difference [RR = 0.99 (95% CI, 0.89–1.11), *p* = 0.87]; at the same time, there was no significant difference in cardiovascular mortality [RR = 1.17 (95% CI, 0.98–1.40),*p* = 0.08] or all-cause mortality [RR = 1.03 (95% CI, 0.91–1.17),*p* = 0.62]. In terms of 2-stage meta-analyses, there was no significant difference in any outcomes at any time point (moderate-to low-certainty evidence).

**Conclusion:** In patients without atherosclerotic disease, febuxostat likely has a similar cardiovascular profile to allopurinol. However, in patients with a history of cardiovascular disease, allopurinol treatment is associated with less cardiovascular mortality as compared with febuxostat.

**Systematic Review Registration:** https://www.crd.york.ac.uk/prospero/#loginpage, identifier PROSPERO, CRD42022325656.

## 1 Introduction

Gout is a metabolic disease, caused by elevation of serum urate level (Scuiller et al., 2020). The prevalence of gout in the world ranges from 0.68%–3.90% and is still increasing steadily (Dalbeth et al., 2021). Previous evidence showed that gout is a risk factor which can lead to cardiovascular disease (Krishnan et al., 2006; Kuo et al., 2010; Clarson et al., 2015a; Clarson et al., 2015b; Mouradjian et al., 2020). It is common that patients with gout also suffer from cardiovascular disease, and about 74% of patients have hypertension, 10% had a history of stroke, and 14% have a history of myocardial infarction (Zhu et al., 2012). In addition, the risk of death in patients with gout may be increased because of cardiovascular disease (Choi and Curhan, 2007). According to clinical guidelines in many countries, febuxostat and allopurinol are recommended as first-line drugs for treatment of gout (Yamanaka, 2011; Hui et al., 2017; Richette et al., 2017; FitzGerald et al., 2020). Allopurinol, a xanthine oxidase inhibitor, is considered one of the most effective uric acid-lowering drugs and is often used to treat chronic gout (Seth et al., 2014). Febuxostat reduces uric acid production by effectively and selectively inhibiting two forms of xanthine oxidase. With the approval of febuxostat in 2009, clinicians have a wider selection of drugs to treat gout (Bardin and Richette, 2019).

According to published randomized controlled trials, febuxostat is a more effective option than allopurinol (Becker et al., 2005). However, in 2017 and 2019, the U.S. Food and Drug Administration (FDA) issued two warnings, indicating that febuxostat might increase cardiovascular mortality and all-cause mortality compared with allopurinol in patients with gout (FDA, 2017; FDA, 2019). In addition, two randomized controlled trials with large sample size and long follow-up that focused on the cardiovascular safety of febuxostat and allopurinol received inconsistent conclusions (White et al., 2018; Mackenzie et al., 2020). Previous meta-analysis indicated that allopurinol prevents cardiovascular disease in patients with gout (van der Pol et al., 2021); however, any potential difference in cardiovascular safety between febuxostat and allopurinol should be interpreted. So, in this meta-analysis, we focused on time-event data which evaluated the cardiovascular safety of febuxostat and allopurinol using reconstructed individual-patient data.

## 2 Methods

We followed the PRISMA-IPD (Preferred Reporting Items for Systematic reviews and Meta-Analyses of individual participant data) when carrying out this research and reported the results (Stewart et al., 2015). We registered this study in PROSPERO (CRD42022325656).

### 2.1 Literature search and eligible criteria

With a combination of keywords (gout; allopurinol; febuxostat; drug therapy; randomized controlled trials), we searched PubMed, Embase, CBM, CNKI, WanFang, Central, and VIP comprehensively from inception to 30 January 2022 for relevant studies. In addition, we also searched ClinicalTrials.gov from inception to 30 January 2022 for unpublished data and screened reference lists of eligible studies to identify potential eligible studies.

The inclusion criteria: 1) participants: adult patients (>18 years) with gout. 2) Interventions: febuxostat. 3) Comparison: allopurinol. 4) Outcomes: MACE (major adverse cardiovascular events; a composite endpoint of cardiovascular death, non-fatal myocardial infarction, non-fatal stroke, and urgent revascularization for unstable angina), cardiovascular death, and all-cause death. 5) Study design: randomized controlled trials with Kaplan–Meier curves and had a follow-up of at least 52 weeks.

The exclusion criteria: 1) asymptomatic hyperuricemia, acute gout, and secondary gout. 2) Studies published in a language which is not Chinese or English. 3) Studies with missing data and studies with outcomes other than MACE incidence, cardiovascular mortality, and all-cause mortality. 4) Patients with moderate or severe hepatic impairment (value, ascites, lower limb edema, icterus, and alanine aminotransferase (ALT) or aspartate aminotransferase (AST) > 3× reference or increased prothrombin time >2× reference value). 5) Patients with severe renal impairment (eGFR <15 ml/min). 6) Patients with diseases that seriously affect the outcome indicators (such as immune diseases, hematological diseases, malignant tumors, etc*.*).

### 2.2 Screening process, data extraction, and risk of bias

First, two researchers (XG and SZ) searched databases according to keywords and imported literature into EndNote and then browsed titles and abstracts roughly according to the inclusion and exclusion criteria. For potentially relevant studies, we downloaded the full text of the literature and then read it carefully to decide whether to include it or not. After all the remaining literatures were screened, the entire process is drawn into a flowchart and displayed in the results. Any discrepancies in the screening process will be resolved through the intervention of the third researcher (NS).

Two reviewers (XG and SZ) used R 4.1.3 to extract data from Kaplan–Meier curves in two randomized controlled trials (RCTs) and then reconstructed individual patient-based data (IPD) using an R package *IPDfromKM* (Guyot et al., 2012; Lee et al., 2020).

The study used the revised Risk of Bias 2.0 to evaluate the risk of bias (Sterne et al., 2019). Two members (XG and SZ) independently assessed the risk of bias according to the evaluation method in the tool. After assessment, they cross-checked and made a three-line table to display the results. Any disagreements were resolved by consultation with the third investigator (NS).

### 2.3 Certainty of evidence assessment

Using the GRADE (Grading of Recommendations Assessment, Development, and Evaluation) framework, two authors assessed the certainty of evidence based on five domains (risk of bias, inconsistency, imprecision, publication bias, and indirectness) and then rated the certainty for each outcome as high, moderate, low, or very low (Guyatt et al., 2008; Zeng et al., 2021).

### 2.4 Statistical analysis

First, we performed 1-stage meta-analyses by the reconstructed IPD to evaluate the qualitative trend of the relative effects over time. Risk ratios (RRs) were used to summarize time-to-event outcomes (that is, MACE (major adverse cardiovascular events; a composite endpoint of cardiovascular death, non-fatal myocardial infarction, non-fatal stroke, and urgent revascularization for unstable angina), cardiovascular death, and all-cause death] and calculated by using the multi-level flexible hazard regression model (Tierney et al., 2007). *p* values were calculated using the log-rank test (Bland JM, 2004). The result will be presented as Kaplan–Meier curves.

In addition, using the reconstructed IPD, we also performed 2-stage meta-analyses to evaluate the quantitative estimates of time-specific relative risks at the six time points (1, 2, 3, 4, 5, and 6 years) and robustness of the results. All analyses were completed using the R 4.1.3 (meta-package), and the results will be presented as forest plots.

### 2.5 Role of the funding source

The study design, data collection, data synthesis, and analysis or interpretation were not influenced by funding sources.

## 3 Results

### 3.1 Characteristics of eligible studies

**FIGURE 1:**
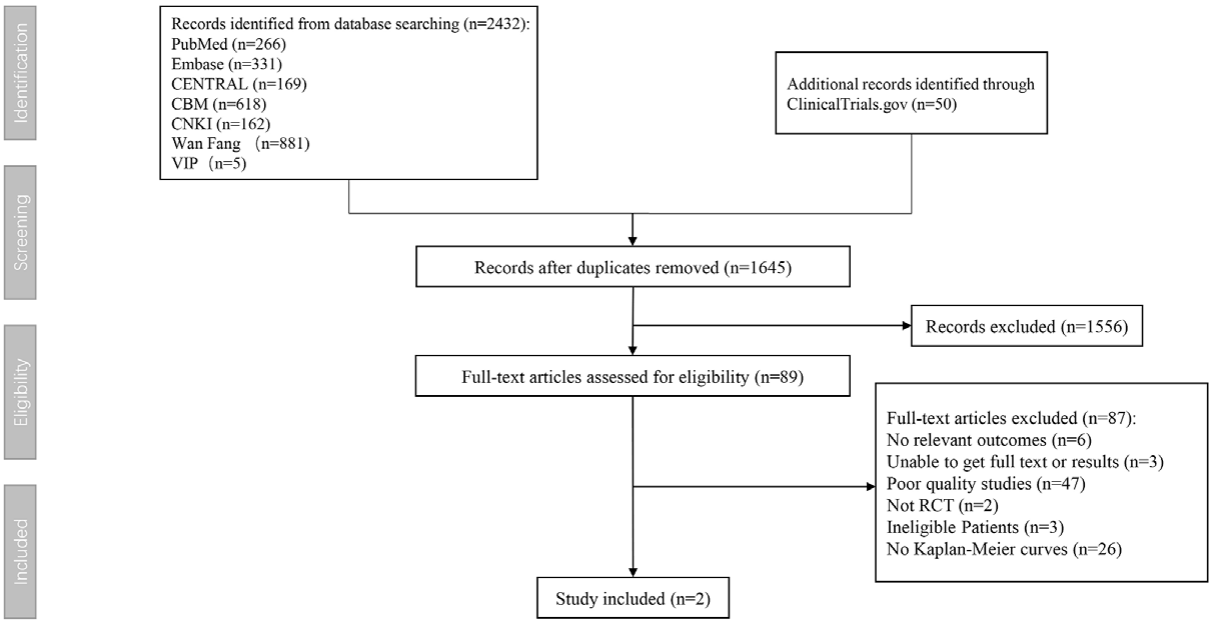
Flow diagram of literature search and selection.

According to the inclusion criteria, we found two eligible randomized controlled trials totaling 12,318 participants in our systematic review (Figure 1). The two inclusion trials were the febuxostat versus allopurinol streamlined trial (FAST) and the cardiovascular safety of febuxostat and allopurinol in patients with gout and cardiovascular morbidities (CARES) trial. The patients in the two trials were all gout patients with cardiovascular comorbidities.

In FAST, 6,128 patients were left in an intention-to-treat analysis (3,063 in the febuxostat group and 3,065 in the allopurinol group) and were followed for a median of 1,467 days (IQR1029-2052). The primary composite endpoint was the first occurrence of hospitalization for non-fatal myocardial infarction or biomarker-positive acute coronary syndrome; non-fatal stroke (whether reported to have led to hospitalization or not or to have occurred during a hospitalization); or death due to a cardiovascular event. The conclusion is that the cardiovascular safety of the two drugs has no statistical difference.

In CARES, 6,190 patients were assigned randomly to receive febuxostat (*n* = 3,098) or allopurinol (*n* = 3,092), and median follow-up time was 32 months (maximum, 85 months). The primary outcome was a composite of cardiovascular death, non-fatal myocardial infarction, non-fatal stroke, or urgent revascularization for unstable angina. The conclusion is that the cardiovascular safety of febuxostat is better than that of allopurinol.

The baseline characteristics of the included studies are summarized in Table 1.

**TABLE 1:** Baseline characteristics of each included study (*n* = 2).

Author (year)	Number (F/A)	Patient	Male proportion (%)	Age		Intervention		Follow-up time	Baseline serum uric acid	Outcome
				F	A	F	A			
White 2018 (CARES)	3,098/3,092	Patients with gout and cardiovascular disease	83.94	64.0, (58.0, and 71.0)	65.0, (58.0, and 71.0)	40 mg/day–80 mg/day	300 mg/day–600 mg/day	Median 136 weeks; maximum 364 weeks	0.518 mmol/L	①②③
Mackenzie 2020 (FAST)	3,063/3,065	Patients with gout	85.26	71.0 ± 6.4	70.9 ± 6.5	80 mg/day–120 mg/day	100 mg/day –900 mg/day	Median follow-up time was 1,467 days	0.297 mmol/L	①②③

F, febuxostat; A, allopurinol; ①, all adverse cardiovascular events during follow-up and treatment (a composite endpoint of cardiovascular death, non-fatal myocardial infarction, non-fatal stroke, and urgent revascularization for unstable angina). ② Cardiovascular death (death due to cardiovascular causes during follow-up and treatment). ③ All-cause death (death due to any cause during follow-up and treatment).

### 3.2 Risk of bias of included studies

According to ROB 2, one study (FAST) was evaluated at high risk of bias in the domain of the randomization process, and the other study (CARES) was evaluated at low risk of bias in all domains (Table 2).

**TABLE 2:** Risk of bias assessment results.

Study	R	D	Mi	Me	S	O
Low risk of bias White (2018)	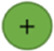	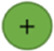	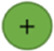	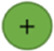	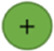	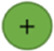
High risk of bias Mackenzie (2020)	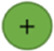	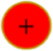	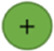	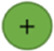	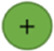	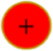

R: bias arising from the randomization process; D: bias due to deviations from intended interventions; Mi: bias due to missing outcome data; Me: bias in measurement of the outcome; S: bias in selection of the reported result; O: overall risk of bias. 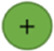: Low risk of bias; 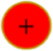: High risk of bias.

### 3.3 Results of 1-stage meta-analysis

**FIGURE 2:**
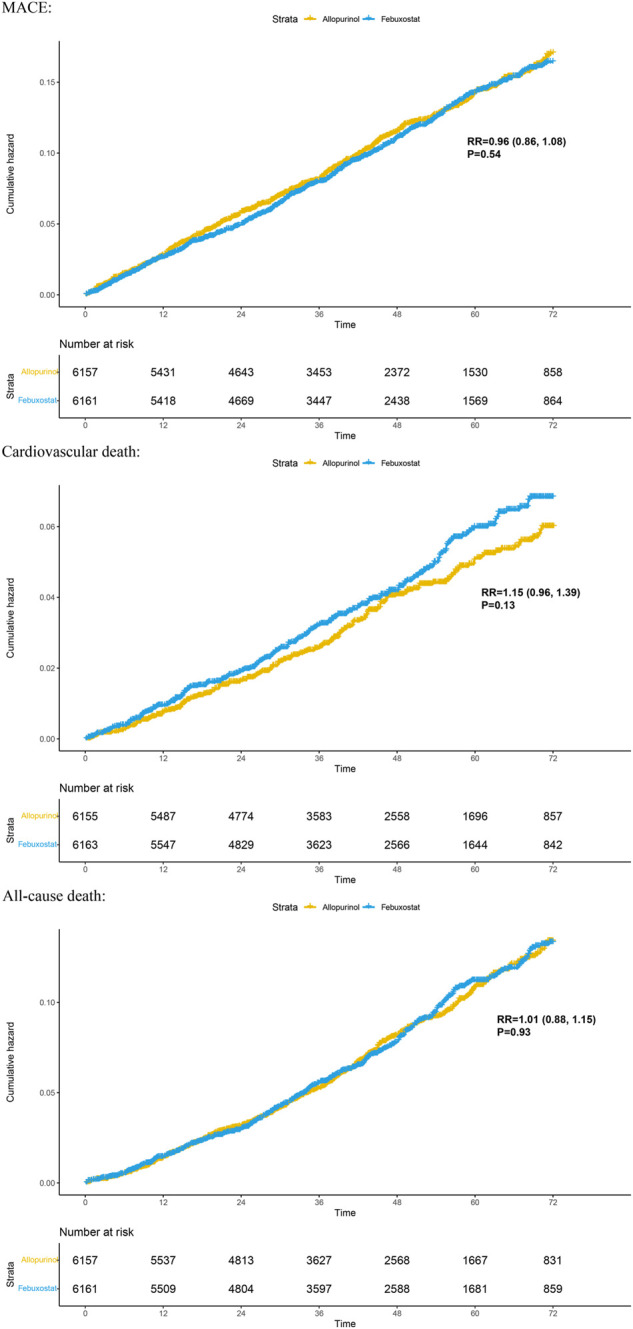
Kaplan–Meier plots for benefit outcomes in 1-stage meta-analyses. In the Kaplan–Meier curves, the ordinate represents the incidence of adverse events, and the abscissa represents time. Two curves with different colors represent different groups; blue represents the febuxostat group, and yellow represents the allopurinol group. The numbers below the curves represent the numbers at risk in different groups at different time points. MACE, major adverse cardiovascular events.

Two randomized controlled trials (including 12,318 patients) provided Kaplan–Meier curves in the study. In the incidence of major adverse cardiovascular events between the two regimens, there was no significant difference [RR = 0.99 (95% CI, 0.89–1.11), *p* = 0.87]; at the same time, there was no significant difference in cardiovascular mortality [RR = 1.17 (95% CI, 0.98–1.40),*p* = 0.08] or all-cause mortality [RR = 1.03 (95% CI, 0.91–1.17),*p* = 0.62]. The curve fitting results are shown in Figure 2.

### 3.4 Results of 2-stage meta-analysis

The results suggested that febuxostat was not associated with a statistically significant increase at all times in the risk of MACE, cardiovascular mortality, and all-cause mortality (moderate-to low-certainty evidence). In cardiovascular mortality, we found significant heterogeneity at 5 years (I2 = 53%, *p* = 0.14) and 6 years (I2 = 70%, *p* = 0.07). In all-cause mortality, we found significant heterogeneity at 3 years (I2 = 61%, *p* = 0.11), 4 years (I2 = 80%, *p* = 0.02), 5 years (I2 = 84%, *p* = 0.01), and 6 years (I2 = 88%, *p* < 0.01). Because of heterogeneity between two RCTs, we used random-effects models. (Table 3 and Appendix Figure 3).

**TABLE 3:** GRADE profiles: febuxostat compared to allopurinol for gout.

Outcome	Illustrative comparative risks* (95% CI)		Relative effect (95% CI)	No of participant (studies)	Quality assessment						Quality of the evidence (GRADE)
	Assumed risk	Corresponding risk			Design	Risk of bias	Inconsistency	Indirectness	Imprecision	Other consideration	
	Allopurinol	Febuxostat									
**All adverse cardiovascular events (1 year)**	**Study population**		**RR 0.95**	12,318 (two studies)	Randomized trials	Serious^1^	No serious inconsistency	No serious indirectness	No serious imprecision	None	⊕⊕⊕⊝ **Moderate** ^1^
	**26 per 1,000**	25 per 1,000 (19 to 32)	(0.73–1.23)								
**All adverse cardiovascular events (2 years)**	**Study population**		**RR 0.86**	12,318 (two studies)	Randomized trials	Serious^1^	No serious inconsistency	No serious indirectness	No serious imprecision	None	⊕⊕⊕⊝ **Moderate** ^1^
	**51 per 1,000**	44 per 1,000 (37 to 51)	(0.73–1)								
**All adverse cardiovascular events (3 years)**	**Study population**		**RR 0.96**	12,318	Randomized trials	Serious^1^	No serious inconsistency	No serious indirectness	No serious imprecision	None	⊕⊕⊕⊝ **Moderate** ^1^
	**66 per 1,000**	63 per 1,000 (55 to 72)	(0.84–1.1)								
**All adverse cardiovascular events (4 years)**	**Study population**		**RR 0.95**	12,318	Randomized trials	Serious^1^	No serious inconsistency	No serious indirectness	No serious imprecision	None	⊕⊕⊕⊝ **Moderate** ^1^
	**82 per 1,000**	78 per 1,000 (67 to 90)	(0.82–1.1)								
**All adverse cardiovascular events (5 years)**	**Study population**		**RR 0.97**	12,318 (two studies)	Randomized trials	Serious^1^	No serious inconsistency	No serious indirectness	No serious imprecision	None	⊕⊕⊕⊝ **Moderate** ^1^
	**91 per 1,000**	95 per 1,000 (83 to 110)	(0.87–1.09)								
**All adverse cardiovascular events (6 years)**	**Study population**		**RR 0.97**	12,318 (two studies)	Randomized trials	Serious^1^	Serious^2^	No serious indirectness	No serious imprecision	None	⊕⊕⊝⊝ **Low** ^1^ ^,^ ^2^
	**98 per 1,000**	95 per 1,000 (83 to 110)	(0.84–1.12)								
**Cardiovascular death (1 year)**	**Study population**		**RR 1.25**	12,318 (two studies)	Randomized trials	Serious^1^	No serious inconsistency	No serious indirectness	No serious imprecision	None	⊕⊕⊕⊝ **Moderate** ^1^
	**7 per 1,000**	9 per 1,000(6 to 13)	(0.84–1.85)								
**Cardiovascular death (2 years)**	**Study population**		**RR 1.13**	12,318 (two studies)	Randomized trials	Serious^1^	No serious inconsistency	No serious indirectness	No serious imprecision	None	⊕⊕⊕⊝ **Moderate** ^1^
	**11 per 1,000**	26 per 1,000 (21 to 33)	(0.82–1.55)								
**Cardiovascular death (3 years)**	**Study population**		**RR 1.25**	12,318	Randomized trials	Serious^1^	No serious inconsistency	No serious indirectness	No serious imprecision	None	⊕⊕⊕⊝ **Moderate** ^1^
	**21 per 1,000**	26 per 1,000 (21 to 33)	(0.99–1.57)								
**Cardiovascular death (4 years)**	**Study population**		**RR 1.09**	12,318 (two studies)	Randomized trials	Serious^1^	No serious inconsistency	No serious indirectness	No serious imprecision	None	⊕⊕⊕⊝ **Moderate** ^1^
	**28 per 1,000**	31 per 1,000 (25 to 38)	(0.87–1.36)								
**Cardiovascular death (5 years)**	**Study population**		**RR 1.17**	12,318 (two studies)	Randomized trials	Serious^1^	No serious inconsistency^3^	No serious indirectness	No serious imprecision	None	⊕⊕⊕⊝ **Moderate** ^1^ ^,^ ^3^
	**32 per 1,000**	37 per 1,000 (28 to 48)	(0.89–1.53)								
**Cardiovascular death (6 years)**	**Study population**		**RR 1.13**	12,291 (two studies)	Randomized trials	Serious^1^	No serious inconsistency^4^	No serious indirectness	No serious imprecision	None	⊕⊕⊕⊝ **Moderate** ^1^ ^,^ ^4^
	**36 per 1,000**	41 per 1,000 (30 to 56)	(0.82–1.55)								
**All-cause death (1 year)**	**Study population**		**RR 1.02**	12,318	Randomized trials	Serious^1^	No serious inconsistency	No serious indirectness	No serious imprecision	None	⊕⊕⊕⊝ **Moderate** ^1^
	**13 per 1,000**	14 per 1,000 (10 to 18)	(0.76–1.38)								
**All-cause death (2 years)**	**Study population**		**RR 0.94**	12,318 (two studies)	Randomized trials	Serious^1^	No serious inconsistency	No serious indirectness	No serious imprecision	None	⊕⊕⊕⊝ **Moderate** ^1^
	**28 per 1,000**	26 per 1,000 (21 to 33)	(0.75–1.2)								
**All-cause death (3 years)**	**Study population**		**RR 1.03**	12,318 (two studies)	Randomized trials	Serious^1^	Serious^5^	No serious indirectness	No serious imprecision	None	⊕⊕⊝⊝ **Low** ^1^ ^,^ ^5^
	**42 per 1,000**	44 per 1,000 (33 to 57)	(0.79–1.35)								
**All-cause death (4 years)**	**Study population**		**RR 0.98**	12,318 (two studies)	Randomized trials	Serious^1^	Serious^6^	No serious indirectness	No serious imprecision	None	⊕⊕⊝⊝ **Low** ^1^ ^,^ ^6^
	**51 per 1,000**	50 per 1,000 (35 to 72)	(0.69–1.4)								
**All-cause death (5 years)**	**Study population**		**RR 1.02**	12,318 (two studies)	Randomized trials	Serious^1^	Serious^3^	No serious indirectness	No serious imprecision	None	⊕⊕⊝⊝ **Low** ^1^ ^,^ ^3^
	**66 per 1,000**	76 per 1,000 (53 to 109)	(0.73–1.42)								
**All-cause death (6 years)**	**Study population**		**RR 1.01**	12,318 (two studies)	Randomized trials	Serious^1^	Serious^4^	No serious indirectness	No serious imprecision	None	⊕⊕⊝⊝ **Low** ^1^ ^,^ ^4^
	**75 per 1,000**	76 per 1,000 (53 to 109)	(0.71–1.45)								

* The basis for the **assumed risk** (e.g., the median control group risk across studies) is provided in footnotes. The **corresponding risk** (and its 95% confidence interval) is based on the assumed risk in the comparison group and the **relative effect** of the intervention (and its 95% CI); **CI:** confidence interval; **RR:** risk ratio; **moderate quality(**⊕⊕⊕⊝**):** further research is likely to have an important impact on our confidence in the estimate of effect and may change the estimate; and **low quality (**⊕⊕⊝⊝**):** further research is very likely to have an important impact on our confidence in the estimate of effect and is likely to change the estimate.

1 Downgraded one level for risk of bias (Mackenize et al., 2020: high risk of bias for blinding).

2 Downgraded one level for inconsistency (substantial heterogeneity was present among the studies (I2 = 44%, *p* = 0.18). One study’s conclusion contradicted another’s).

3 Downgraded one level for inconsistency (substantial heterogeneity was present among the studies (I2 = 84%, *p* = 0.01). One study’s conclusion contradicted another’s).

4 Downgraded one level for inconsistency (substantial heterogeneity was present among the studies (I2 = 88%, *p* < 0.01). One study’s conclusion contradicted another’s).

5 Downgraded one level for inconsistency (substantial heterogeneity was present among the studies (I2 = 61%, *p* = 0.11). One study’s conclusion contradicted another’s).

6 Downgraded one level for inconsistency (substantial heterogeneity was present among the studies (I2 = 80%, *p* = 0.02). One study’s conclusion contradicted another’s).

**FIGURE 3:**
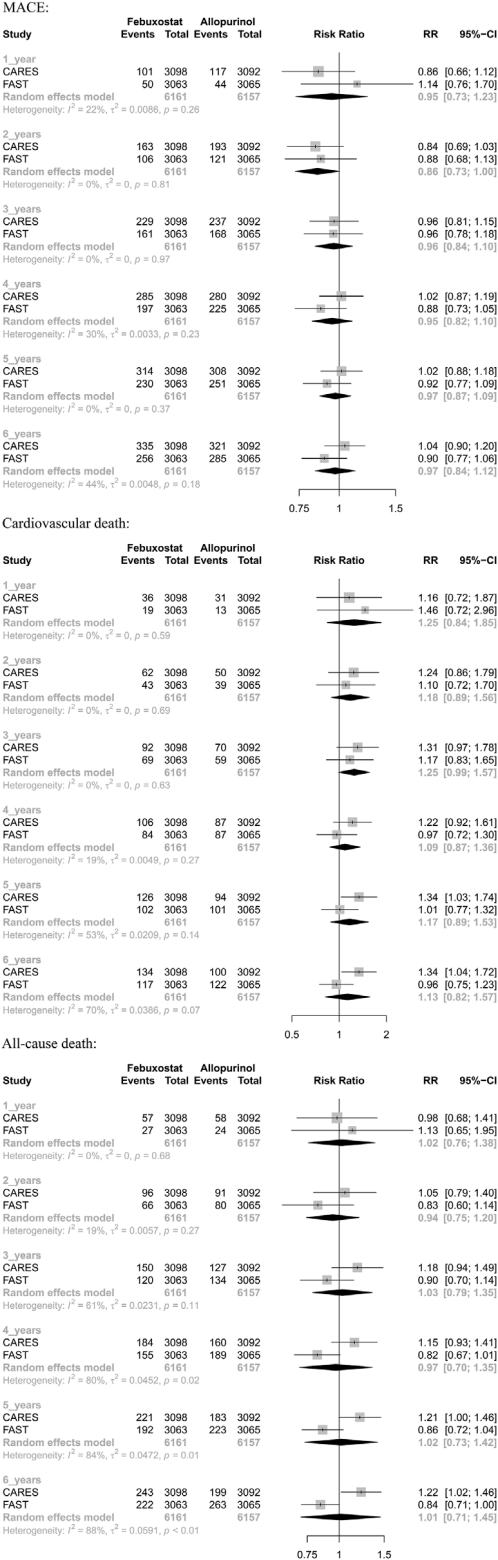
Forest plots of time-specific relative risks in 2-stage meta-analyses.

## 4 Discussion

To compare the cardiovascular safety of febuxostat and allopurinol in patients with gout, we conducted 1-stage meta-analysis based on reconstructed individual patient data and 2-stage analysis at different time points. The result indicates that, compared to allopurinol, febuxostat does not increase the incidence of MACE, cardiovascular death, or all-cause death in the treatment of patients with gout.

For heterogeneity between the two studies in two-stage meta-analysis, we speculate the following reasons: 1) the baseline characteristics are different in two trials, such as the proportion of patients with cardiovascular disease (in CARES, almost 40% of the study population has a history of myocardial infarction, 14% a history of stroke, and around 12% a history of peripheral artery disease, while these percentages were considerably lower in the FAST trial: 10%, 5%, and 5%, respectively). Because the reconstructed IPD may not completely represent the indeed IPD, these differences in baseline prevalence of cardiovascular disease between FAST and CARES may potentially affect the cardiovascular outcomes; 2) doses of medicines are different. In CARES, the dose of allopurinol is 200–600 mg/day, and the dose of febuxostat is 40–80 mg/day, and in FAST, the dose of allopurinol is 100–900 mg/day, and the dose of febuxostat is 80–120 mg/day. It is worth considering that the risk of adverse drug events usually increases with increasing drug dose; however, the lower dose of febuxostat in CARES increases all-cause mortality and cardiovascular mortality than that in FAST. Therefore, we believe that the result of FAST, which is consistent with our conclusion, is more reliable; 3) the loss rate of CARES is higher than that of FAST; 4) differences in sponsors, practitioners, and trial procedures may also lead to differences in final conclusions. However, considering that the two RCTs both met the inclusion and exclusion criteria, the sample sizes were both sufficient, and the follow-up time met the requirements. Hence, we do not think that the stability of the results will be affected. As a method which evaluates the robustness of 1-stage meta-analysis, our results of 2-stage meta-analysis showed consistent results.

In addition to the two randomized controlled trials, there exist other studies about the cardiovascular safety of febuxostat and allopurinol, and the conclusions are also inconsistent. Above all, our conclusion is consistent with that of one network meta-analysis (Zhang et al., 2021), three systematic meta-analyses (Liu et al., 2019; Barrientos-Regala et al., 2020; Gao et al., 2021), and two cohort studies (Chen et al., 2019; Kang et al., 2019). However, our findings are inconsistent with those of one cohort study in the real world (Su et al., 2019). Considering that even if the study used relevant statistical methods to minimize the impact of covariates on outcome indicators, it still cannot be considered that all possible covariates have been dealt with, and the research results still need to be corroborated by randomized controlled trials with high data quality or real-world data. Therefore, we believe that our research results are still reliable, which can provide specific reference significance for clinical practice and provide a certain basis for the selection of XOI drugs for clinical treatment of gout.

Our research not only enriches the content of related fields but also provides a certain reference for the selection of uric acid-lowering drugs for the clinical treatment of gout. Our meta-analysis has the following advantages: 1) to the best of our knowledge, individual-patient data level meta-analysis was not used to compare the cardiovascular safety of febuxostat and allopurinol in patients with gout before, and our study is the first to adopt this approach. 2) This 1-stage meta-analysis presents the results as Kaplan–Meier curves, which can reflect the time-event more intuitively and can visually observe the comparison of cardiovascular safety at various time points.

The main limitations of our study are the following: 1) the inclusion criteria were not so strict, so some patients with various diseases were included in this study, which may have resulted in some heterogeneity or bias. However, this study can still give clinical references for treatment of gout because gout patients in the real world often have comorbid diseases. 2) Because the language is limited to Chinese and English, some studies may be omitted. 3) Only two studies were included, and this problem may be solved by more published relevant randomized controlled trials or real-world studies.

## 5 Conclusion

Febuxostat likely has a similar cardiovascular profile to allopurinol in patients without atherosclerotic disease based on the reconstructed IPD. However, in patients with a history of cardiovascular disease, allopurinol treatment is associated with less cardiovascular mortality as compared with febuxostat. Because their results are inconclusive, febuxostat still needs to be used cautiously for patients with gout and cardiovascular diseases.

## Data Availability

The original contributions presented in the study are included in the article/Supplementary Materials; further inquiries can be directed to the corresponding author.

## References

[B1] Bardin T. Richette P. (2019). The role of febuxostat in gout. Curr. Opin. Rheumatol. 31, 152–158. 10.1097/BOR.0000000000000573 30601228

[B2] Barrientos-Regala M. Macabeo R. A. Ramirez-Ragasa R. PestañO N. S. Punzalan F. E. R. Tumanan-Mendoza B. (2020). The association of febuxostat compared with allopurinol on blood pressure and major adverse cardiac events among adult patients with hyperuricemia: A meta-analysis. J. Cardiovasc. Pharmacol. 76, 461–471. 10.1097/FJC.0000000000000871 32675751

[B3] Becker M. A. Schumacher H. R. JR. Wortmann R. L. Macdonald P. A. Eustace D. Palo W. A. (2005). Febuxostat compared with allopurinol in patients with hyperuricemia and gout. N. Engl. J. Med. 353, 2450–2461. 10.1056/NEJMoa050373 16339094

[B4] Bland Jm A. D. Altman D. G. (2004). The logrank test. BMJ 328 (7447), 1073. 10.1136/bmj.328.7447.1073 15117797 PMC403858

[B5] Chen C. H. Chen C. B. Chang C. J. Lin Y. J. Wang C. W. Chi C. C. (2019). Hypersensitivity and cardiovascular risks related to allopurinol and febuxostat therapy in asians: A population-based cohort study and meta-analysis. Clin. Pharmacol. Ther. 106, 391–401. 10.1002/cpt.1377 30690722

[B6] Choi H. K. Curhan G. (2007). Independent impact of gout on mortality and risk for coronary heart disease. Circulation 116, 894–900. 10.1161/CIRCULATIONAHA.107.703389 17698728

[B7] Clarson L. E. Chandratre P. Hider S. L. Belcher J. Heneghan C. Roddy E. (2015a). Increased cardiovascular mortality associated with gout: A systematic review and meta-analysis. Eur. J. Prev. Cardiol. 22, 335–343. 10.1177/2047487313514895 24281251 PMC4361356

[B8] Clarson L. E. Hider S. L. Belcher J. Heneghan C. Roddy E. Mallen C. D. (2015b). Increased risk of vascular disease associated with gout: A retrospective, matched cohort study in the UK clinical practice research datalink. Ann. Rheum. Dis. 74, 642–647. 10.1136/annrheumdis-2014-205252 25165032 PMC4392302

[B9] Dalbeth N. Gosling A. L. Gaffo A. Abhishek A. (2021). Gout. Lancet 397, 1843–1855. 10.1016/S0140-6736(21)00569-9 33798500

[B10] FDA (2019). Adds boxed warning for increased risk of death with gout medicine uloric (febuxostat). [Online]. Available at: https://www.fda.gov/drugs/drug-safety-and-availability/fda-adds-boxed-warning-increased-riskdeath-gout-medicine-uloric-febuxostat (Accessed 2 21, 2019).

[B11] FDA (2017). FDA to evaluate increased risk of heart-related death and death from all causes with the gout medicine febuxostat (Uloric). Available: https://www.fda.gov/drugs/drug-safety-and-availability/fda-drug-safety-communication-fda-evaluate-increased-risk-heart-related-death-and-death-all-causes (Accessed 11 15, 2017).

[B12] Fitzgerald J. D. Dalbeth N. Mikuls T. Brignardello-Petersen R. Guyatt G. Abeles A. M. (2020). 2020 American college of rheumatology guideline for the management of gout. Arthritis Rheumatol. 72, 879–895. 10.1002/art.41247 32390306

[B13] Gao L. Wang B. Pan Y. Lu Y. Cheng R. (2021). Cardiovascular safety of febuxostat compared to allopurinol for the treatment of gout: A systematic and meta-analysis. Clin. Cardiol. 44, 907–916. 10.1002/clc.23643 34013998 PMC8259158

[B14] Guyatt G. H. Oxman A. D. Vist G. E. Kunz R. Falck-Ytter Y. Alonso-Coello P. (2008). Grade: An emerging consensus on rating quality of evidence and strength of recommendations. BMJ 336, 924–926. 10.1136/bmj.39489.470347.AD 18436948 PMC2335261

[B15] Guyot P. Ades A. E. Ouwens M. J. Welton N. J. (2012). Enhanced secondary analysis of survival data: Reconstructing the data from published kaplan-meier survival curves. BMC Med. Res. Methodol. 12, 9. 10.1186/1471-2288-12-9 22297116 PMC3313891

[B16] Hui M. Carr A. Cameron S. Davenport G. Doherty M. Forrester H. (2017). The British society for rheumatology guideline for the management of gout. Rheumatol. Oxf. 56, 1246–1059. 10.1093/rheumatology/kex250 28605531

[B17] Kang E. H. Choi H. K. Shin A. Lee Y. J. Lee E. B. Song Y. W. (2019). Comparative cardiovascular risk of allopurinol versus febuxostat in patients with gout: A nation-wide cohort study. Rheumatol. Oxf. 58, 2122–2129. 10.1093/rheumatology/kez189 31098635

[B18] Krishnan E. Baker J. F. Furst D. E. Schumacher H. R. (2006). Gout and the risk of acute myocardial infarction. Arthritis Rheum. 54, 2688–2696. 10.1002/art.22014 16871533

[B19] Kuo C. F. See L. C. Luo S. F. Ko Y. S. Lin Y. S. Hwang J. S. (2010). Gout: An independent risk factor for all-cause and cardiovascular mortality. Rheumatol. Oxf. 49, 141–146. 10.1093/rheumatology/kep364 19933595

[B20] Lee J. J. Liu N. Zhou Y. (2020). IPDfromKM: Map digitized survival curves back to individual patient data. *R package version 0.1.10.* [Online]. Available: https://CRAN.R-project.org/package=IPDfromKM (Accessed 11 11, 2020). 10.1186/s12874-021-01308-8 PMC8168323 34074267

[B21] Liu C. W. Chang W. C. Lee C. C. Shau W. Y. Hsu F. S. Wang M. L. (2019). The net clinical benefits of febuxostat versus allopurinol in patients with gout or asymptomatic hyperuricemia - a systematic review and meta-analysis. Nutr. Metab. Cardiovasc. Dis. 29, 1011–1022. 10.1016/j.numecd.2019.06.016 31378626

[B22] Mackenzie I. S. Ford I. Nuki G. Hallas J. Hawkey C. J. Webster J. (2020). Long-term cardiovascular safety of febuxostat compared with allopurinol in patients with gout (FAST): A multicentre, prospective, randomised, open-label, non-inferiority trial. Lancet 396, 1745–1757. 10.1016/S0140-6736(20)32234-0 33181081

[B23] Mouradjian M. T. Plazak M. E. Gale S. E. Noel Z. R. Watson K. Devabhakthuni S. (2020). Pharmacologic management of gout in patients with cardiovascular disease and heart failure. Am. J. Cardiovasc. Drugs 20, 431–445. 10.1007/s40256-020-00400-6 32090301

[B24] Richette P. Doherty M. Pascual E. Barskova V. Becce F. CastañEDA-Sanabria J. (2017). 2016 updated EULAR evidence-based recommendations for the management of gout. Ann. Rheum. Dis. 76, 29–42. 10.1136/annrheumdis-2016-209707 27457514

[B25] Scuiller A. Pascart T. Bernard A. Oehler E. (2020). Gout. Rev. Med. Interne 41, 396–403. 10.1016/j.revmed.2020.02.014 32201015

[B26] Seth R. Kydd A. S. Buchbinder R. Bombardier C. Edwards C. J. (2014). Allopurinol for chronic gout. Cochrane Database Syst. Rev. 2014, Cd006077. 10.1002/14651858.CD006077 PMC8915170 25314636

[B27] Sterne J. A. C. Savović J. Page M. J. Elbers R. G. Blencowe N. S. Boutron I. (2019). RoB 2: A revised tool for assessing risk of bias in randomised trials. Bmj 366, l4898. 10.1136/bmj.l4898 31462531

[B28] Stewart L. A. Clarke M. Rovers M. Riley R. D. Simmonds M. Stewart G. (2015). Preferred reporting items for systematic review and meta-analyses of individual participant data: The PRISMA-IPD statement. Jama 313, 1657–1665. 10.1001/jama.2015.3656 25919529

[B29] Su C. Y. Shen L. J. Hsieh S. C. Lin L. Y. Lin F. J. (2019). Comparing cardiovascular safety of febuxostat and allopurinol in the real world: A population-based cohort study. Mayo Clin. Proc. 94, 1147–1157. 10.1016/j.mayocp.2019.03.001 31272565

[B30] Tierney J. F. Stewart L. A. Ghersi D. Burdett S. Sydes M. R. (2007). Practical methods for incorporating summary time-to-event data into meta-analysis. Trials 8, 16. 10.1186/1745-6215-8-16 17555582 PMC1920534

[B31] Van der pol K. H. Wever K. E. Verbakel M. Visseren F. L. J. Cornel J. H. Rongen G. A. (2021). Allopurinol to reduce cardiovascular morbidity and mortality: A systematic review and meta-analysis. PLoS One 16, e0260844. 10.1371/journal.pone.0260844 34855873 PMC8638940

[B32] White W. B. Saag K. G. Becker M. A. Borer J. S. Gorelick P. B. Whelton A. (2018). Cardiovascular safety of febuxostat or allopurinol in patients with gout. N. Engl. J. Med. 378, 1200–1210. 10.1056/NEJMoa1710895 29527974

[B33] Yamanaka H. (2011). Japanese guideline for the management of hyperuricemia and gout: Second edition. Nucleosides Nucleotides Nucleic Acids 30, 1018–1029. 10.1080/15257770.2011.596496 second edition 22132951

[B34] Zeng L. Brignardello-Petersen R. Hultcrantz M. Siemieniuk R. A. C. Santesso N. Traversy G. (2021). GRADE guidelines 32: GRADE offers guidance on choosing targets of GRADE certainty of evidence ratings. J. Clin. Epidemiol. 137, 163–175. 10.1016/j.jclinepi.2021.03.026 33857619

[B35] Zhang S. Xu T. Shi Q. Li S. Wang L. An Z. (2021). Cardiovascular safety of febuxostat and allopurinol in hyperuricemic patients with or without gout: A network meta-analysis. Front. Med. 8, 698437. 10.3389/fmed.2021.698437 PMC8239361 34211992

[B36] Zhu Y. Pandya B. J. Choi H. K. (2012). Comorbidities of gout and hyperuricemia in the US general population: Nhanes 2007-2008. Am. J. Med. 125, 679–687. 10.1016/j.amjmed.2011.09.033 22626509

